# Heavy alcohol use exacerbates skeletal myopathy in peripheral artery disease

**DOI:** 10.1007/s11357-025-01965-3

**Published:** 2025-11-12

**Authors:** Panagiotis Koutakis, Emma Fletcher, Evlampia Papoutsi, William T. Bohannon, Iraklis I. Pipinos, Dimitrios Miserlis, Pal Pacher

**Affiliations:** 1 Department of Cellular and Integrative Physiology, University of Nebraska at Medical Center, Omaha, NE USA; 2 Department of Surgery, University of Nebraska Medical Center, Omaha, NE USA; 3 Department of Surgery, Baylor Scott and White Medical Center, Temple, TX USA; 4 Department of Surgery, University of Texas at Austin Dell Medical School, Austin, TX USA; 5 Laboratory of Cardiovascular Physiology and Tissue Injury, National Institute on Alcohol Abuse and Alcoholism, National Institutes of Health, Bethesda, MD USA

**Keywords:** Chronic alcohol misuse, Peripheral artery disease, Myopathy, Oxidative stress, Aldehyde dehydrogenase-2

## Abstract

Peripheral artery disease (PAD) is an age-associated vascular condition that contributes to progressive skeletal muscle dysfunction, marked by oxidative stress, mitochondrial abnormalities, and muscle atrophy. Chronic alcohol misuse independently induces similar myopathic changes. Whether alcohol use disorder (AUD) worsens PAD-related skeletal muscle pathology in humans remains unknown. We analyzed gastrocnemius biopsies from PAD patients with heavy alcohol misuse (PAD-AUD), PAD patients without heavy alcohol misuse (PAD-LA), and non-PAD controls with low-to-no alcohol consumption (CON). Markers of oxidative stress, mitochondrial respiration and myofiber morphometrics were compared across groups. Lower extremity function—including walking distance and ankle plantar flexor strength—was also evaluated. Markers of muscle oxidative stress (4-hydroxynonenal (4-HNE), protein carbonyls, and acetaldehyde), mitochondriopathy and myofiber morphometric damage, were significantly elevated in PAD-LA patients relative to CON. Functional outcomes, including walking ability and muscle strength, were correspondingly reduced in PAD. Notably, these pathological and functional impairments were further exacerbated in PAD-AUD. Interestingly, levels of the alcohol-detoxifying enzyme aldehyde dehydrogenase 2 (ALDH2) were unchanged in PAD-AUD muscle but were significantly upregulated in PAD-LA. Although traditionally recognized for metabolizing alcohol-derived aldehydes, ALDH2 also facilitates the clearance of endogenous reactive aldehydes generated by mitochondrial oxidative stress, including 4-HNE and protein carbonyls. In PAD-LA patients, elevated ALDH2 levels coincided with reduced oxidative stress and mitochondriopathy, whereas PAD-AUD patients exhibited no ALDH2 upregulation and intramuscular pathology was enhanced. These findings implicate ALDH2 dysfunction as a contributing factor to muscle degeneration in PAD-AUD and support its potential as a therapeutic target for attenuating oxidative stress in PAD.

**Graphical abstract:**

**Figure:**
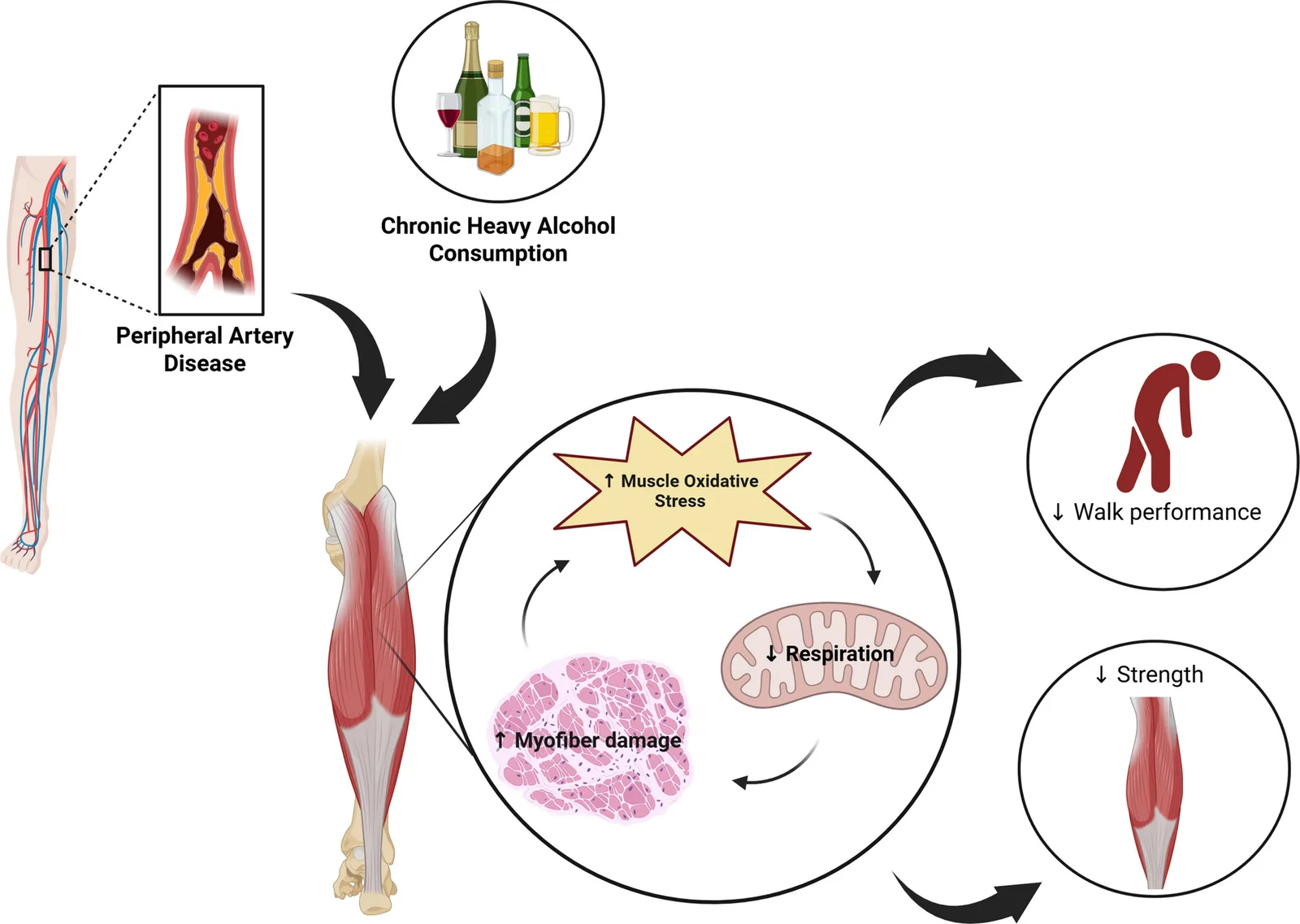

**Supplementary Information:**

The online version contains supplementary material available at https://doi.org/10.1007/s11357-025-01965-3.

## Introduction

Peripheral artery disease (PAD) is an age-associated vascular condition that contributes substantially to morbidity and mortality through progressive ischemic muscle damage [1–3]. This PAD-mediated myopathy is characterized by intramuscular oxidative stress, mitochondrial dysfunction, and structural remodeling, including myofiber atrophy and fibrosis [3–27]. While aging is a central risk factor, growing evidence implicates heavy chronic alcohol consumption as a compounding contributor to PAD pathogenesis [28, 29]. Beyond its known capacity to accelerate vascular injury and atherogenesis [30, 31], heavy alcohol consumption has also been associated with impaired mitochondrial function, increased generation of reactive oxygen and nitrogen species (ROS/RNS) [32], and dysregulation of endothelial, vascular, and cardiac homeostasis [33]. Such maladaptations may synergistically accelerate cardiovascular aging and exacerbate ischemic myopathy in PAD by further compromising skeletal muscle integrity and perfusion.

Chronic alcohol misuse is a well-documented and independent contributor to skeletal muscle pathology, with an incidence that exceeds that of alcohol-associated liver cirrhosis by at least fivefold [34–37], and is associated with substantial reductions in quality of life and increased mortality risk [38, 39]. Notably, the pathological features of alcohol-induced myopathy—characterized by elevated oxidative stress, mitochondrial dysfunction, and muscle fiber atrophy—closely mirror those observed in PAD-associated myopathy [34–36, 38, 40–42]. In a recent proof-of-concept study, it has been demonstrated that prolonged ethanol exposure exacerbated skeletal muscle degeneration in aged mice with chronic hindlimb ischemia [43]. However, whether a similar intramuscular pathological signature is present in PAD patients with comorbid alcohol use disorder (AUD) remains to be determined.

Given the rising prevalence of alcohol misuse in both the general and aging populations [44–46], and reports of disproportionately high alcohol consumption among individuals with PAD [47], the primary objective of this study was to evaluate skeletal muscle morphometrics, mitochondrial function, and oxidative damage in gastrocnemius biopsies from PAD patients with alcohol use disorder (AUD), compared to PAD patients and non-PAD controls consuming low-to-moderate or no alcohol. Recent evidence highlights aldehyde dehydrogenase 2 (ALDH2), a mitochondrial enzyme involved in alcohol metabolism, as a key regulator of oxidative stress and mitochondrial function [48–52]. Our group previously demonstrated that ALDH2 expression is significantly reduced in the skeletal muscle of high-fat-fed, alcohol-exposed mice with hindlimb ischemia, coinciding with increased oxidative stress and mitochondrial dysfunction [43]. Building on these findings, the second aim of this study was to determine whether ALDH2 expression is similarly altered in the gastrocnemius muscle of PAD patients with AUD.

## Methods

### Participants

Data from PAD patients and healthy adults were used from an existing biobank of samples (PK). The protocol for the collection of such specimens was approved by the University of Texas at Austin Dell Medical School (CR-23–058) and the Baylor Scott and White Medical Center (402,270). We specifically selected 56 patients with symptomatic PAD and 30 non-PAD control patients with a known medical history of alcohol consumption. A PAD diagnosis was based on medical history, physical examination, reduced ankle-brachial index (ABI) at rest (< 0.90), and computerized or standard arteriography. Non-PAD control patients presented to the medical clinics for indications other than PAD, had normal lower extremity pulses and blood flow, normal ABIs at rest and after stress, no history of PAD symptoms, and all led a sedentary lifestyle. Individuals with musculoskeletal or neurologic symptoms unrelated to PAD, or acute lower extremity ischemic events secondary to thromboembolic disease or trauma, were excluded from the study.

### Alcohol consumption

Habitual alcohol use was determined from medical history information and patient interviews with medical personnel. Using the definition of the National Institute on Alcohol Abuse and Alcoholism (NIAAA) [53], 28 PAD patients were classified as heavy drinkers/with AUD (i.e., consumed > 14 or > 7 drinks/week, for males and females, respectively). An additional 28 PAD patients and 30 non-PAD healthy adults consumed moderate-to-low or no alcohol and served as experimental controls for this study.

### Functional assessments

The six-minute walk test was used to assess lower extremity function. The test was performed indoors in a long, flat, straight 20-m hallway, and the total distance walked over a 6-min timeframe (6MWD) was recorded in meters [54]. A Gardner graded exercise treadmill test was also used to determine the absolute claudication distance (ACD); i.e., the distance at which claudication pain became intolerable, forcing the patient to stop walking [55, 56]. In the non-PAD controls, data for the ACD variable represents the distance corresponding to exercise cessation due to volitional fatigue rather than exertional leg pain. Muscle strength was measured using an isometric dynamometer (Biodex System 4.0; Biodex Medical Systems, Shirley, NY). Isometric contraction of the ankle plantar flexor muscle peak force was measured during a maximum isometric contraction held for 10 s, and the data are represented in N*m [25, 26].

### Muscle biopsy

For the PAD patients with intermittent claudication (IC) and the non-PAD controls, biopsies were obtained from the anteromedial aspect of the gastrocnemius, 10 cm distal to the tibial tuberosity via fine needle (12G) aspiration. Gastrocnemius samples were acquired during lower leg amputation in patients with critical limb ischemia (CLI) and taken from the same anatomical region described for the IC and non-PAD control patient samples. Approximately 250 mg muscle was obtained from all patients. For histological analyses, a portion of the biopsy was immediately placed in cold methacarn, transferred to cold ethanol:H_2_O (50:50 v/v) after 48 h, and subsequently embedded in paraffin [19, 25, 26, 57]. For mitochondrial measures, another portion of the sample was immediately placed in fresh ice-cold relaxation solution (10 mM EGTA, 3 mM free Mg^2+^, 20 mM taurine, 0.5 mM DTT, 20 mM imidazole, 5 mM ATP, 15 mM phosphocreatine, and 160 mM K-MES, pH 7.2) and transferred to the lab for respiration assays [12]. The remaining tissue was snap-frozen in liquid nitrogen for all other analyses.

### Histology and quantitative fluorescence microscopy

Muscle biopsy specimens embedded in paraffin were sectioned at 4 microns and labeled with fluorescent reagents to quantify markers of oxidative damage; carbonyl groups and the Michael adduct of hydroxynonenal (4-HNE), as previously described [5, 19, 25]. Briefly, individual myofibers were identified by sarcolemma labeling with 10 µg/mL Alexa Fluor 647 conjugated Wheat Germ Agglutinin (ThermoFisher, Waltham, MA, W32466). For protein carbonyl staining, endogenous biotin groups were first blocked and then carbonyl groups were biotinylated overnight at 4 °C by treating the slide specimens with 5 mM biocytin-hydrazide (Setareh Biotech, Eugene, OR, 6769). The slides were subsequently labeled with Alexa Fluor 488 conjugated streptavidin (10 µg/mL) (ThermoFisher, S32354) for 1 h at room temperature. To assess 4-HNE adducts, a monoclonal mouse antibody (20 µg/mL) (R&D Systems, Minneapolis, MN, MAB3249) was applied to slide specimens and incubated overnight at 4 °C. The slides were then labeled with Alexa Fluor 594 conjugated anti-mouse IgG (10 µg/mL) (ThermoFisher, A11005) for 1 h at room temperature. All fluorescent-labeled specimens were mounted in ProLong Gold anti-fade medium with DAPI nuclear stain (ThermoFisher, P36931) prior to image acquisition.

### Image acquisition and analysis

Quantification of inter-myofiber carbonyls and 4-HNE adducts was based on three-channel imaging of microscopic fields, with channels corresponding to 1) the sarcolemma, 2) carbonyl groups, and 3) 4-HNE adducts. Fluorescence imaging was captured with the 10 × objective of an epifluorescence microscope (EVOS M7000, ThermoFisher). Celleste software (ThermoFisher) was used for data acquisition and analysis. Several microscopic fields were captured per specimen, corresponding to 3,500–15,000 myofibers. The fluorescent signal within each myofiber was corrected for background and expressed as the mean pixel intensity in grayscale units (gsu) on a 12-bit gray scale, which corresponded to the concentration of carbonyls or 4-HNE within the myofiber [5, 19, 25]. The mean fluorescence signal from all myofibers present in each specimen was determined.

### Morphometric measures

Morphometric parameters of myofiber size and shape were determined from the Wheat Germ Agglutinin-marked myofibers as previously described in detail [10, 26]. Cross-sectional area (in square microns) was assessed as the number of pixels enclosed within a segmented myofiber, diameter (in microns) was defined as the diameter of a circle with the same area as the segmented myofiber region, and roundness was determined as the equivalent diameter x Π, divided by the perimeter of the segmented region [10].

### Mitochondrial respirometry

Mitochondrial respiration was evaluated in saponin-permeabilized muscle fibers using an Oroboros O2K high-resolution fluororespirometer (Oroboros Instruments, Austria) [13, 43, 58]. Four separate assays were performed to assess respiration at each complex of the electron transport chain (ETC). Complex I-dependent respiration was measured by the addition of 5 mM glutamate, 5 mM malate, and 1 mM ADP; Complex II assessments used 1 mM ADP, 10 mM succinate, and 3 μM rotenone (to inhibit complex I); Complex III required 3 μM rotenone, 1 mM ADP, and 1 mM duroquinol; and Complex IV respiration was assessed using 3 μM rotenone, 1 mM ADP, 0.2 mM N,N,N,N-tetramethyl-p-phenylenediamine (TMPD), and 10 mM ascorbate to prevent TMPD auto-oxidation [59]. The respiration rates were then normalized to citrate synthase (CS) activity (nanoatomO_2_/min/unit CS activity) as a proxy for intramuscular mitochondrial content.

CS activity was determined colorimetrically via the reduction of the substrate 5,5′-Dithiobis-(2-nitrobenzoic acid) (DTNB) by coenzyme A (CoA-SH) and recorded as the increase in absorbance at a wavelength of 412 nm (DU Series 640 spectrophotometer, Beckman Instruments, Fullerton, CA). CS activity was then normalized to sample protein concentration (µmol/min/mg protein) determined using a Pierce BCA assay (Thermo Scientific, Rockford, IL, 23,225) as previously described [2, 59].

### Real time polymerase chain reaction

Total RNA was isolated from frozen muscle biopsy samples using the Direct-zol™ RNA Microprep Kit (Zymo Research, Irvine, CA, R2063) and RNA concentration and quality was determined using a NanoDrop One spectrophotometer (Thermo Scientific). RNA purity was accepted if the A260/A280 ratio was between 1.8–2.1. The RNA was then reverse transcribed to cDNA using the iScript™ Reverse Transcription Supermix for RT-qPCR Kit (Bio-Rad, Hercules, CA, 1,708,841). PCR reactions used the following target genes of the ETC: NADH:ubiquinone oxidoreductase subunit B8 (NDUFB8, complex I; qHsaCEP0040853), Succinate Dehydrogenase Complex Iron Sulfur Subunit B (SDHB, complex II; qHsaCID0011704), Ubiquinol-Cytochrome C Reductase Core Protein 2 (UQCRC2, complex III; qHsaCIP0033016), Cytochrome c oxidase (COX10, complex IV; qHsaCEP0049189), ATP synthase F1 subunit alpha (ATP5A1, complex V; qHsaCEP0025064), as well as target genes for alcohol-metabolizing enzymes: alcohol dehydrogenase 1B (ADH1B; qHsaCEP0039662) and ALDH2 (qHsaCEP0050628). The housekeeping gene was glyceraldehyde-3-phosphate dehydrogenase (GAPDH; qHsaCED0038674). All primers were obtained from and validated by Bio-Rad Laboratories (Bio-Rad Laboratories, Hercules, CA, 10,025,220).

PCR reactions were run on a CFX Opus Real-Time PCR System (Bio-Rad, Hercules, CA) using SsoAdvanced Universal SYBR Green Supermix (Bio-Rad, 1,725,271). The quantification cycle (Cq) of all target genes was normalized to the housekeeping gene (GAPDH). The relative normalized expression (the delta delta Ct value) was then calculated using the 2^(-delta delta Cq) method [60]. All samples were run in triplicate, and results were averaged.

### Protein expression analysis by western blot

Protein was extracted from frozen muscle samples using an ice-cold lysis buffer containing 50 mM Tris–HCl, pH 8.0, 150 mM NaCl, 1% triton X-100, 0.5% sodium-deoxycholate, 0.1% sodium dodecyl sulfate, and 1 × protease inhibitor cocktail (Sigma Aldrich, Burlington, MA, P8340) and the protein content within the muscle homogenate was determined using a BCA assay (Thermo Scientific, 23,225). Following the addition of 2 × Laemmli sample buffer (Bio-Rad Laboratories, 1,610,747) and 2-mercaptoethanol reducing agent (Bio-Rad Laboratories, 1,610,710), sample protein (20 µg) was separated using electrophoresis in a Criterion Cell Tank (Bio-Rad Laboratories) and 4–20% Criterion TGX Precast Midi Protein Gels (Bio-Rad Laboratories, 5,671,095). Proteins were then transferred to 0.2 µm Amersham Hybond P polyvinylidene difluoride (PVDF) transfer membranes (Cytiva, Malborough, MA, 10,600,021) and total protein per lane was established using Ponceau S stain (Boston BioProducts, Milford, MA, ST180). The membranes were subsequently incubated with the following primary antibodies: 4-HNE (1:1000, Abcam, Waltham, MA, ab46545), OxiSelect Protein Carbonyl anti-DNP antibody (1:1000, Cell Biolabs, Inc, San Diego, CA, STA-308), ALDH2 (1:500, Proteintech, Rosemont, IL, 15,310–1-AP), ADH1B (1:1000, Proteintech, 66,939–1-Ig), and OXPHOS human antibody cocktail (1:200, Thermo Scientific, 45–8199). Secondary antibodies used included Goat anti-Rabbit IgG (1:10,000, Invitrogen, Waltham, MA, 31,462) or Goat anti-Mouse IgG (1:10,000, Invitrogen, 31,430). Band visualization and detection was achieved using Clarity ECL Substrate (Bio-Rad, 1,705,060) and the ChemiDoc MP Imaging System (Bio-Rad Laboratories). The band intensity of each target protein was quantified using Image Lab software (Version 6.1, Bio-Rad Laboratories) and normalized to sample total protein (Ponceau S stain). The protein content of each target is expressed as mean fold change relative to non-PAD control muscle ± SD.

### Colorimetric measures of oxidative stress

Acetaldehyde accumulation: An acetaldehyde assay kit (Sigma Aldrich, Burlington, MA, MAK434) was used to quantify acetaldehyde content in patient muscle homogenate (20 μg protein) and serum (undiluted). The assay is based on the aldehyde dehydrogenase-catalyzed oxidation of acetaldehyde and reduction of NAD to NADH. The resulting NADH reduces MTT, producing a colored formazan compound detected at 565 nm, which is directly proportional to the sample acetaldehyde concentration. Measurements were performed in duplicate and acetaldehyde content is expressed as mg/dL (serum) or normalized to sample total protein (mg/dL/mg protein) (muscle homogenate).

Superoxide dismutase (SOD) activity: The enzymatic activity of the antioxidant SOD was assessed in the muscle lysates (20 μg protein) using an SOD activity kit (Sigma Aldrich, CS0009) as described in detail elsewhere [58]. All samples, controls, and standards were assayed in duplicate and SOD activity was expressed as units of activity/mL and normalized to total protein within the muscle homogenate (U/mL/mg).

### Statistical analysis

Demographic characteristics of PAD patients with AUD, low/no-alcohol consuming PAD patients and non-PAD control subjects were compared using chi-square or Fisher’s exact tests for categorical variables and one-way analysis of variance (ANOVA) with Tukey post-hoc tests for continuous variables. ANOVA with Tukey post-hoc adjustment was also used to determine group differences in all other biological targets of interest. Prism statistical software (version 10.5.0, GraphPad, Boston, MA) was used to perform the statistical analyses. Data are reported as mean ± SD for continuous variables, and frequencies with proportions for categorical variables, and significance was accepted at *P* < 0.05.

## Results

Demographic information for non-PAD controls, PAD patients with heavy alcohol consumption (PAD-AUD) and PAD patients consuming low/no-alcohol (PAD-LA) are listed in Table 1. Aside from the number of alcoholic beverages consumed per week, PAD-AUD and PAD-LA patients were otherwise matched for PAD severity and all other demographics and comorbidities (*P* > 0.05). Both PAD patient groups showed reduced walking ability compared to controls (*P* < 0.05), which was further reduced in the PAD-AUD group. Ankle plantarflexor muscle strength was similarly reduced.

**Table 1:** Participant demographics

	Non-PAD Controls (*n*=30)	PAD-LA (*n*=28)	PAD-AUD (*n*=28)	*P*-value
Age (y)	58.9 ± 12.0	64.3 ± 9.4	60.9 ± 5.4	0.071
Gender				0.111
Male (%)	66.7	89.0	71.4	
Female (%)	33.3	11.0	25.6	
Race (%)				0.111
White	76.7	64.3	50.0	
Black	10.0	14.3	7.1	
Hispanic	13.3	21.4	42.9	
Alcoholic drinks (number/week)	1.4 ± 1.6	1.7 ± 2.1	24.9 ± 4.8*^, #^	<0.001
BMI (kg/m^2^)	29.5 ± 5.2	29.0 ± 5.8	25.6 ± 4.6*	0.013
Smoking status				0.201
Never smoked (%)	56.7	28.5	32.0	
Current smoker (%)	23.3	43.0	43.0	
Previous smoker (%)	20.0	28.5	25.0	
Coronary artery disease (%)	16.7	28.5	35.7	0.252
Hypertension (%)	56.7	82.1*	64.2	0.030
Dyslipidemia (%)	33.3	75.0*	71.4*	0.002
Statin Use (%)	33.3	85.0*	85.7*	<0.001
Diabetes mellitus (%)	16.7	53.5*	57.1*	0.002
Renal insufficiency (%)	6.7	10.7	25.0	0.111
Ankle-brachial Index (ABI)	1.11 ± 0.08	0.48 ± 0.29*	0.42 ± 0.26*	<0.001
Six-minute walk distance (m)	375.8 ± 39.4	324.4 ± 55.2*	293.8± 53.5*,^#^	<0.001
Absolute claudication distance (m)	815.6 ± 86.7	312.6 ± 104*	176.4 ± 109*,^#^	<0.001
Muscle strength (N*m)	85.9 ± 10.3	49.5 ± 14.1*,^#^	38.1± 14.3*,^#^	<0.001
Serum acetaldehyde (mg/dL)	0.198 ± 0.09	0.242 ± 0.16	0.334 ± 0.24	0.427

*PAD* peripheral artery disease, *AUD* alcohol use disorder, *PAD-LA* PAD patients consuming low/no-alcohol

Asterisks represent between-group post-hoc comparisons, whereby * denotes a significant difference compared to non-PAD control patients

# represents a significant difference between PAD-AUD and PAD-LA (accepted at *P* < 0.05)

Data are mean ± SD for continuous variables and percentage of study population for categorical variables. Oneway ANOVA or chi square tests were performed to assess for between-group differences, respectively

### Myofiber morphology was abnormal in PAD gastrocnemius samples

Myofiber cross-sectional area and diameter were significantly reduced, and roundness was increased in the gastrocnemius of PAD-LA and PAD-AUD patients compared to controls. When the two PAD patient groups were compared, cross-sectional area and diameter were most reduced, and roundness was most increased in those patients with AUD (Table 2).

**Table 2:** Myofiber morphometrics

	Non-PAD Controls	PAD-LA	PAD-AUD	*P*-value
Cross-Sectional Area (μm^2^)	5220 ± 883	4272 ± 683*	3524 ± 718*^, #^	< 0.001
Diameter (μm)	160.2 ± 23.3	74.6 ± 12.0*	58.9 ± 9.4*^, #^	< 0.001
Roundness (ratio)	0.64 ± 0.04	0.81 ± 0.04*	0.84 ± 0.03*^, #^	< 0.001

Data are mean ± SD. One-way ANOVA was used to test between-group differences. * Denotes a significant difference compared to non-PAD control patients, ^#^ represents a significant difference compared to PAD-LA (accepted at *P* < 0.05). *PAD* peripheral artery disease, *AUD* alcohol use disorder, *PAD-LA* PAD patients with limited/no alcohol consumption

### Intramuscular oxidative stress is heightened in PAD patients with AUD

**Fig. 1:**
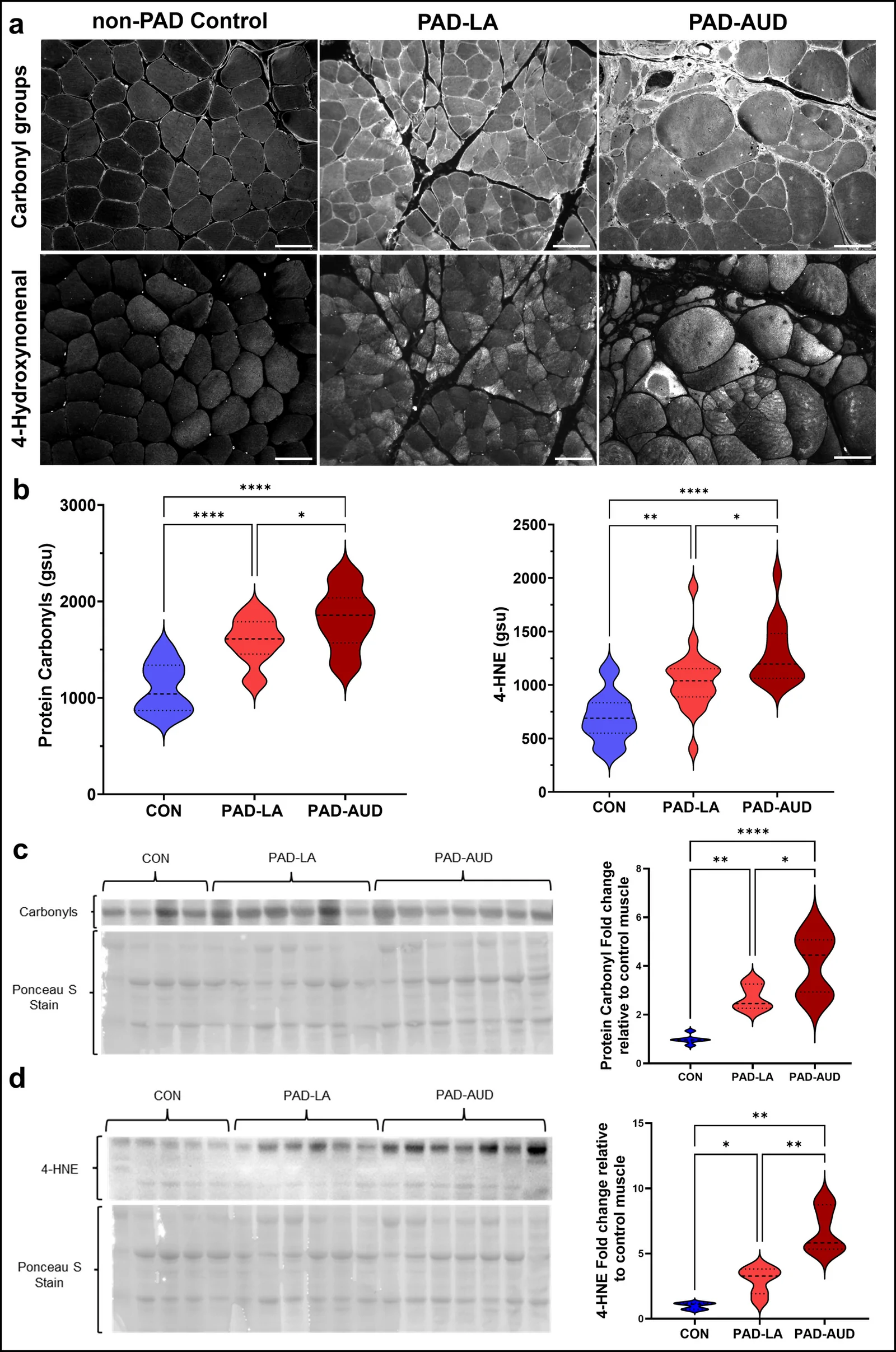
**Muscle oxidative stress is** **exacerbated in PAD patients with comorbid heavy alcohol misuse** (**a**) Representative image of fluorescently labeled protein carbonyl and 4-hydroxynonenol (4-HNE) adducts in gastrocnemius muscle sections from non-PAD controls with low-to-no alcohol consumption (CON), PAD patients without heavy alcohol misuse (PAD-LA), and PAD patients with heavy alcohol misuse (PAD-AUD), all captured with a 10 × objective. The fluorescent signal within each myofiber was corrected for background and expressed as the mean pixel intensity in grayscale units (gsu) on a 12-bit gray scale, which corresponded to the concentration of carbonyls or 4-HNE within the myofiber. (**b**) Quantitative measurements of the mean fluorescence signal for carbonyls and 4-HNE within the myofiber specimens of each patient group. (**c**-**d**) Representative western blots showing protein carbonyl (**c**) and 4-HNE (**d**) content in gastrocnemius muscle homogenate with corresponding fold-change in these adducts. Ponceau S staining represents total protein per lane used for normalization. Asterisks denote * *P* < 0.05, ** *P* < 0.01, **** *P* < 0.0001 for all graphs

**Fig. 2:**
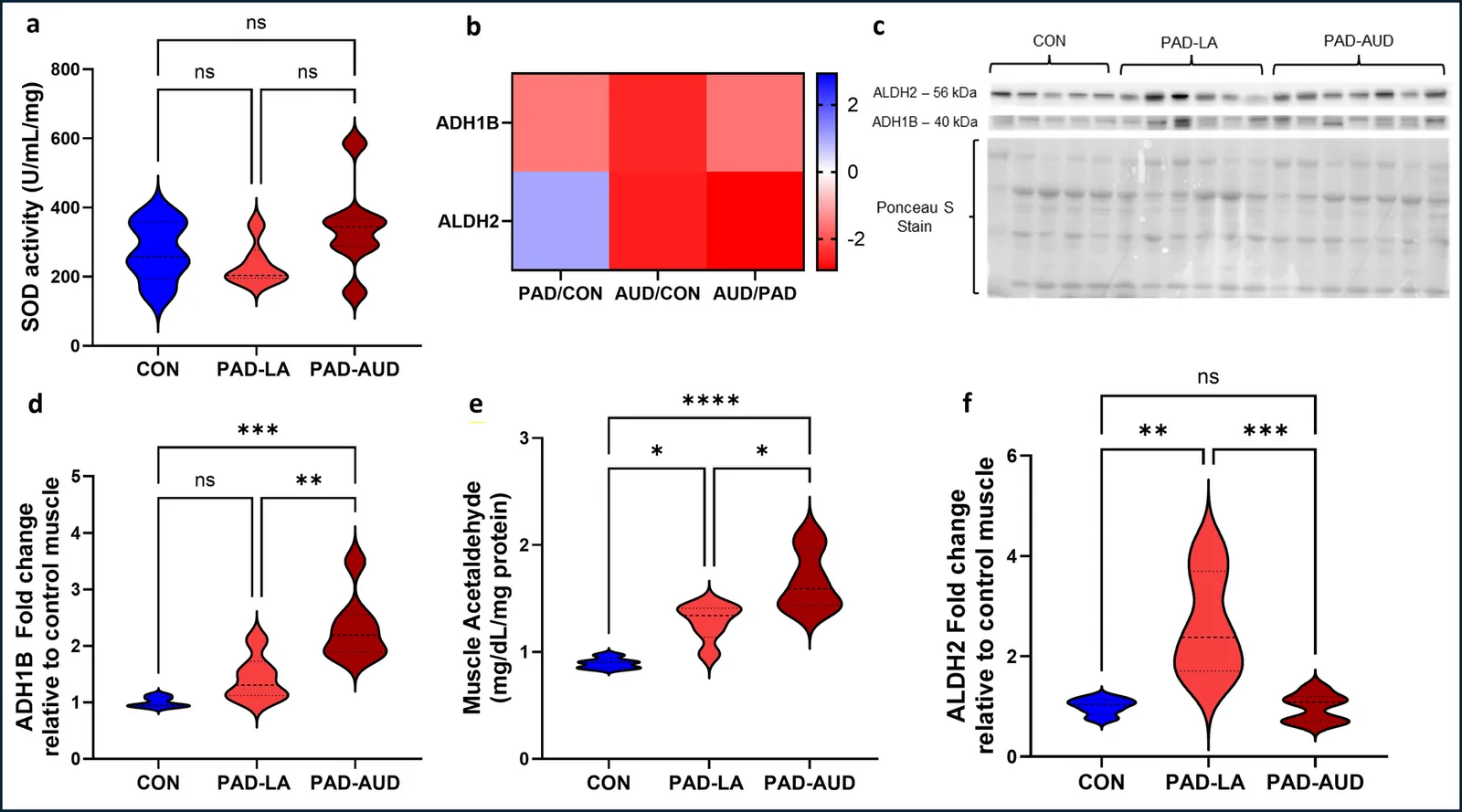
**Intramuscular antioxidant activity is unaltered, yet the content of key alcohol-metabolizing enzymes and their metabolites varies by PAD and degree of alcohol consumption** (**a**) Superoxide dismutase (SOD) enzymatic activity within the gastrocnemius of non-PAD controls with low-to-no alcohol consumption (CON), PAD patients without heavy alcohol misuse (PAD-LA), and PAD patients with heavy alcohol misuse (PAD-AUD). Representative (**b**) heat map and (**c**) western blot showing intramuscular alcohol dehydrogenase 1B (ADH1B) and aldehyde dehydrogenase 2 (ALDH2) gene and protein expression, respectively. Ponceau S staining represents total protein per lane used for normalization. (**d**) Fold-change in gastrocnemius ADH1B protein. (**e**) Intramuscular acetaldehyde concentration. (**f**) Fold-change in gastrocnemius ADH1B protein. Asterisks denote ‘ns’ not significant, * *P* < 0.05, ** *P* < 0.01, *** *P* < 0.001, **** *P* < 0.0001 for all graphs

Histologic measures of muscle oxidative stress, assessed via protein carbonyl accumulation and lipid peroxidation (4-HNE), was significantly greater in PAD patients compared to non-PAD controls (*P* < 0.05) irrespective of patient drinking status (Fig. 1a-b). However, the increase in protein carbonyls and 4-HNE was significantly higher in PAD-AUD compared to PAD-LA (Fig. 1b). Intramuscular protein concentrations of carbonyls and 4-HNE were altered in a similar pattern (Fig. 1c-d). In contrast to 4-HNE and carbonyls, enzymatic activity of the antioxidant SOD was unaltered in PAD-LA (*P* = 0.797) and PAD-AUD (*P* = 0.481) versus controls (Fig. 2a).

Next, we sought to determine whether alcohol-metabolizing enzymes and the oxidative stress-promoting metabolic byproduct, acetaldehyde [34, 36, 38, 42, 61], were altered in PAD. First, despite no significant change in gene expression (*P* > 0.05) (Fig. 2b), ADH1B protein, involved in converting alcohol to acetaldehyde [34, 36, 38, 42], was significantly increased in PAD-AUD muscle (*P* = 0.0004 vs. controls, and *P* = 0.006 vs. PAD-LA) (Fig. 2c-d). Serum acetaldehyde was no different between patient groups (*P* > 0.05) (Table 1). Nevertheless, intramuscular acetaldehyde concentration was increased in PAD-AUD (*P* < 0.0001 and *P* = 0.011 vs. controls and PAD-LA, respectively) (Fig. 2e). Interestingly, muscle acetaldehyde was also elevated in PAD-LA versus non-PAD controls (*P* = 0.019) and coincided with an increase in ALDH2 (*P* = 0.002 vs. controls, and *P* = 0.001 vs. PAD-AUD) (Fig. 2c and f), i.e., the protein responsible for removing acetaldehyde [48–52]. In contrast, ALDH2 gene expression was 2.6-fold (*P* = 0.062) and 2.9-fold (*P* = 0.014) down-regulated in PAD-AUD muscle compared to control and PAD-LA muscle, respectively (Fig. 2b). Although intramuscular ALDH2 protein was also reduced in PAD-AUD patients compared to PAD-LA muscle (*P* = 0.001), protein expression was unaltered compared to controls (*P* = 0.999) (Fig. 2c and f).

### Heavy alcohol consumption worsens PAD-associated declines in mitochondrial function and composition

**Fig. 3:**
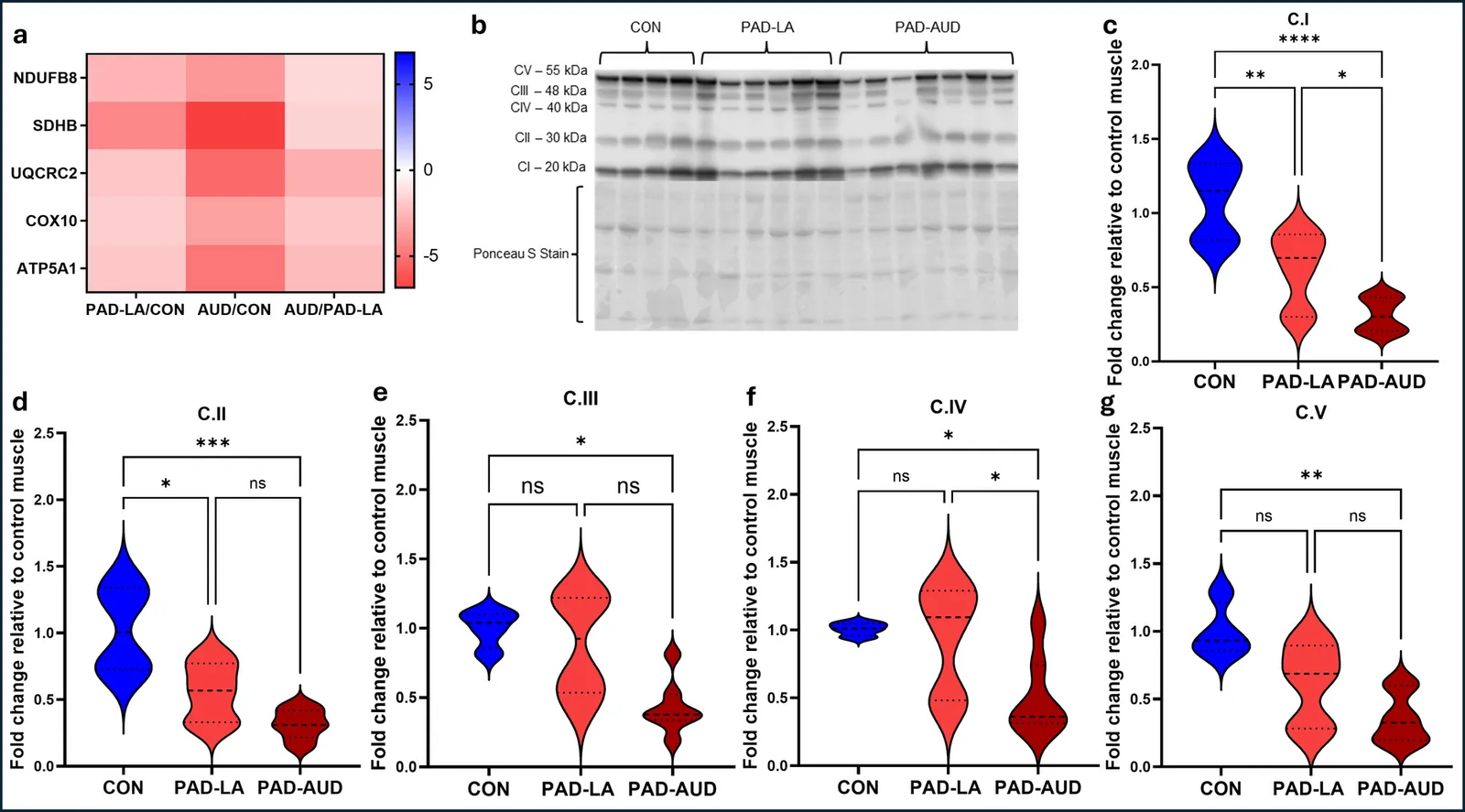
**Mitochondrial electron transport chain (ETC) composition in the gastrocnemius muscle is altered by PAD and alcohol misuse** (**a**) Representative heat map showing the intramuscular fold-change in gene expression for the ETC components NADH:ubiquinone oxidoreductase subunit B8 (NDUFB8, complex I), Succinate Dehydrogenase Complex Iron Sulfur Subunit B (SDHB, complex II), Ubiquinol-Cytochrome C Reductase Core Protein 2 (UQCRC2, complex III), Cytochrome c oxidase (COX10, complex IV) and ATP synthase F1 subunit alpha (ATP5A1, complex V). The heat map columns represent specific between-group gene expression comparisons for each gene target (**b**) Representative western blot and (**c**-**g**) corresponding quantitative measurements of the intramuscular protein content of the individual ETC components (complexes I-IV) as assessed in non-PAD controls with low-to-no alcohol consumption (CON), PAD patients without heavy alcohol misuse (PAD-LA), and PAD patients with heavy alcohol misuse (PAD-AUD). Asterisks denote ‘ns’ not significant, * *P* < 0.05, ** *P* < 0.01, *** *P* < 0.001, **** *P* < 0.0001 for all graphs

**Fig. 4:**
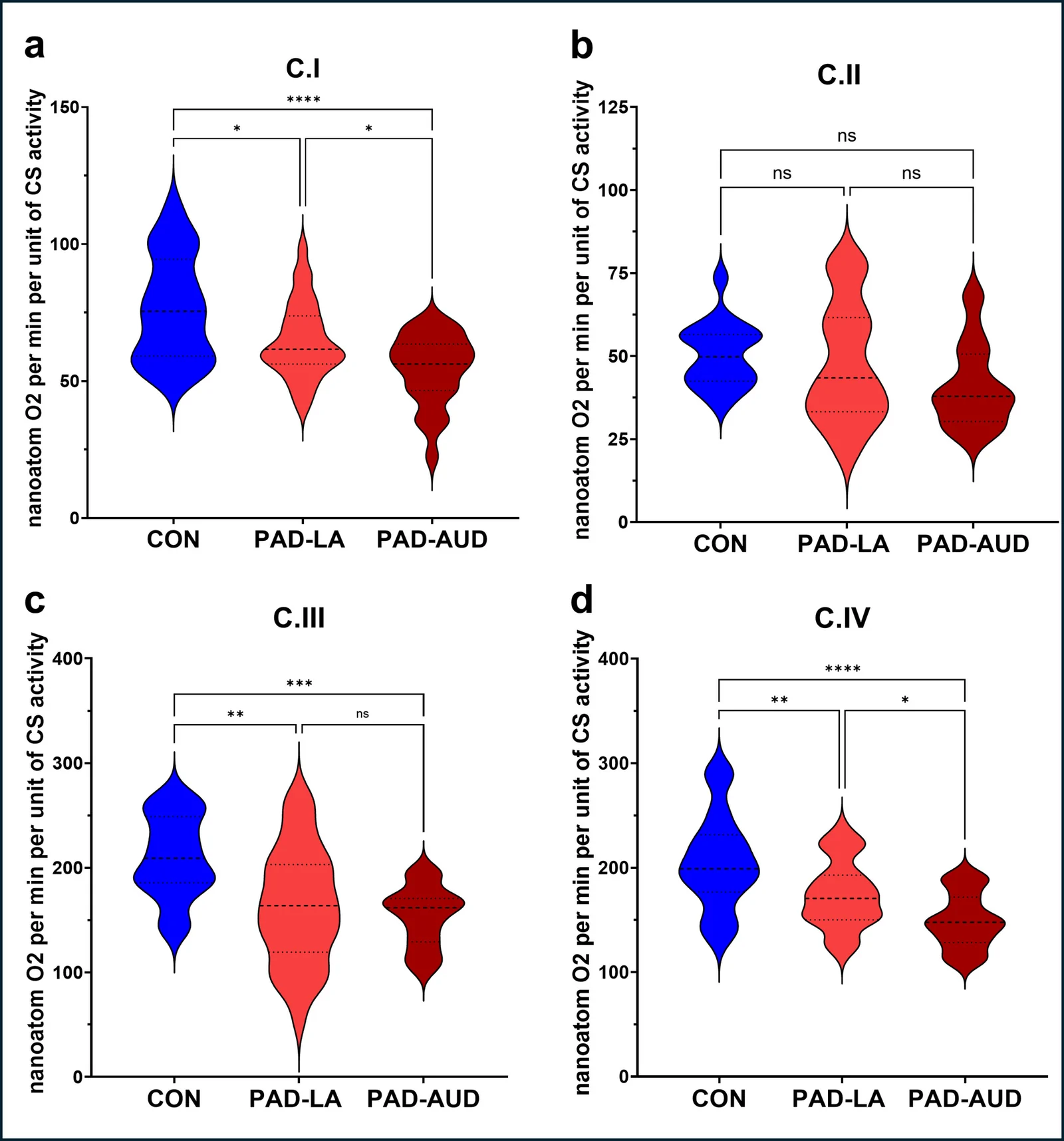
**Heavy alcohol misuse exacerbates PAD-associated declines in intramuscular mitochondrial respiration.** Mitochondrial oxygen (O2) consumption rate in permeabilized gastrocnemius muscle fibers from non-PAD controls with low-to-no alcohol consumption (CON), PAD patients without heavy alcohol misuse (PAD-LA), and PAD patients with heavy alcohol misuse (PAD-AUD). Mitochondrial O2 consumption rate measured during (**a**) Complex I (C.I) respiration, (**b**) Complex II (C.II) respiration, (**c**) Complex III (C.III) respiration and (d) Complex IV (C.IV) respiration. Asterisks denote ‘ns’ not significant, * *P* < 0.05, ** *P* < 0.01, **** *P* < 0.0001

In PAD-LA muscle, gene expression was significantly downregulated compared to control muscle for the genes NDUFB8 (CI; 2.7-fold, *P* = 0.028), SDHB (CII; 4.3-fold, *P* = 0.011), UQCRC2 (CIII; twofold, *P* = 0.038) and ATP5A1 (CV; twofold, *P* = 0.006) (Fig. 3a). However, C.I (*P* = 0.038 vs. control muscle) and C.II (*P* = 0.012 vs. control muscle) were the only ETC complexes showing reduced intramuscular protein abundance in PAD-LA patients (Fig. 4b-d). Conversely, in PAD-AUD muscle, gene down-regulation involved all five genes representing the individual ETC complexes (*P* < 0.05 vs. controls), and when compared to PAD-LA patients, fold down-regulation was significantly greater in PAD-AUD muscle for UQCRC2 (C.III; 2.9-fold, *P* = 0.027), COX10 (C.IV; 2.1-fold, *P* = 0.041) and ATP5A1 (C.V; 2.4-fold, *P* = 0.028). Similarly, ETC protein loss in PAD-AUD patients included all five ETC complexes (*P* < 0.05 vs. control muscle) (Fig. 4b-g). Moreover, the fold-change reduction in C.I and C.IV protein was more extensive in PAD-AUD muscle compared to PAD-LA muscle (C.I: *P* = 0.024, C.IV: *P* = 0.043 vs. PAD-LA) (Fig. 3c and f).

Despite no difference in overall mitochondrial content (CS activity; CON = 132.9 ± 31 vs. PAD-LA:131.7 ± 30 vs. PAD-AUD:133.8 ± 20), mitochondrial respiration revealed significant alterations to ETC complexes (Fig. 4). Compared to gastrocnemius muscle from non-PAD controls, mitochondrial oxygen consumption was significantly lower in both PAD patient groups during ETC Complex I (C.I), Complex III (C.III) and Complex IV (C.IV) respiration (Fig. 3). No differences were observed for Complex II (C.II) respiration. When the two PAD patient groups were compared, respiration was lower in PAD-AUD muscle at C.I (*P* = 0.040) and C.IV (*P* = 0.030).

## Discussion

To our knowledge, this is the first human study to demonstrate that chronic heavy alcohol consumption exacerbates skeletal muscle pathology in patients with PAD. Our findings build on preclinical work from our group showing that long-term ethanol exposure in mice with chronic hindlimb ischemia intensifies intramuscular oxidative stress, lipotoxicity, and mitochondrial dysfunction [43]. In this study, we found that habitual heavy alcohol use in PAD patients (PAD-AUD) was associated with elevated muscle oxidative stress, greater depletion of mitochondrial electron transport chain (ETC) complexes, impaired mitochondrial respiration, and more severe myofiber damage. These structural and bioenergetic changes coincided with functional impairments in walking distance and muscle strength.

Consistent with prior literature, chronic malperfusion in PAD contributes to progressive muscle oxidative stress, mitochondrial dysfunction, and degeneration [3–5, 12, 15–19, 21–24, 26, 27, 62–66]. As expected, these features were evident in both PAD groups, regardless of alcohol use. However, the presence of AUD clearly exacerbated these pathologies, suggesting an additive or synergistic mechanism. A likely contributor is acetaldehyde, a reactive alcohol metabolite that induces substantial oxidative injury. Specifically, acetaldehyde formation during alcohol metabolism generates excess mitochondrial ROS, damaging both structural and functional mitochondrial components [35, 37, 38, 67, 68]. We previously showed that ischemic muscles from alcohol-fed mice exhibited significantly higher H₂O₂ production and acetaldehyde accumulation than non-alcohol-exposed counterparts, coinciding with severe ETC disruption [43]. In the present study, acetaldehyde was markedly higher in PAD-AUD muscle, providing a plausible mechanistic link.

Beyond direct oxidative damage, both acetaldehyde and ROS form covalent adducts with lipids and proteins, producing reactive byproducts like 4-hydroxynonenal (4-HNE) and protein carbonyls [37, 40, 68, 69]. These perpetuate oxidative injury through feed-forward amplification of lipid peroxidation. We found elevated levels of both 4-HNE and protein carbonyls in PAD-AUD muscle, supporting the hypothesis that alcohol-mediated oxidative stress significantly worsens PAD myopathy.

Interestingly, acetaldehyde was also increased in PAD patients who consumed little-to-no alcohol (PAD-LA), albeit to a lesser extent. This is consistent with our animal studies, where ischemic muscle from high-fat-fed mice (without ethanol exposure) accumulated acetaldehyde [43]. A plausible source is malondialdehyde (MDA), a lipid peroxidation byproduct that degrades to acetaldehyde and forms MDA-acetaldehyde adducts—known contributors to cardiovascular pathology even in the absence of alcohol use [70–72]. Given the recognized presence of intramuscular fat in PAD [5, 16, 18, 19, 21, 22, 26, 27, 59], increased lipid peroxidation could explain low-level acetaldehyde accumulation in PAD-LA muscle.

The failure of endogenous antioxidant defenses, notably superoxide dismutase (SOD), to rise in PAD muscle despite elevated oxidative stress suggests possible inhibition by electrophilic species such as ROS and acetaldehyde [49, 61, 70]. While SOD activity was comparable between PAD-AUD and PAD-LA muscle, oxidative biomarkers were markedly higher in PAD-AUD, indicating that other antioxidant systems may be differentially impaired in the context of alcohol misuse.

A key finding was the divergent expression of ALDH2—a mitochondrial enzyme critical for metabolizing acetaldehyde and lipid-derived aldehydes like 4-HNE. ALDH2 was significantly reduced in PAD-AUD muscle, consistent with our murine alcohol model [43], while it was increased in PAD-LA muscle. This suggests that ALDH2 downregulation in AUD may drive aldehyde accumulation and worsen pathology. However, even in PAD-LA patients, elevated ALDH2 failed to fully mitigate oxidative damage, indicating that the response may be inadequate under sustained ischemic stress.

Mechanistically, 4-HNE may mediate ALDH2 inactivation via covalent adduct formation at its active site [73–75]. At high concentrations, 4-HNE inhibits ALDH2 irreversibly and competes with acetaldehyde for binding, impairing both alcohol and lipid aldehyde detoxification [73–75]. This model fits our findings—greater 4-HNE in PAD-AUD may have suppressed ALDH2 activity, while elevated but sub-threshold 4-HNE in PAD-LA allowed a partial ALDH2 response without full functional efficacy.

### Limitations

Several limitations should be acknowledged. First, direct quantification of mitochondrial reactive oxygen species (ROS) was not feasible in this clinical context due to methodological constraints. However, the elevated levels of established oxidative stress markers—4-hydroxynonenal (4-HNE), protein carbonyls, and acetaldehyde—combined with congruent findings from our previous animal models, provide strong indirect evidence of mitochondrial oxidative stress. Nevertheless, other direct quantification assays, such as high-resolution respirometry using Amplex UltraRed reagent and/or tissue imaging with fluorescent probes such as MitoSOX Red [76] could be utilized in the future to further confirm the results of the present study. Second, although we demonstrated clear histopathological and functional impairments associated with AUD in PAD, the limited availability of biopsy material restricted our ability to perform comprehensive molecular analyses of the signaling pathways underlying these changes and to compare the results of our primary outcome measures separately for males and females. Despite these constraints, our study offers important translational insights and establishes a robust platform for future investigations into the molecular mechanisms linking alcohol misuse, aldehyde toxicity, and skeletal muscle dysfunction in PAD.

### Conclusions

With the rising prevalence of alcohol misuse [45, 46], and its recognition as a scientific priority in cardiovascular disease by the American Heart Association, there is a growing need to identify mechanistic links between alcohol use and vascular comorbidities [44]. This study is the first to provide clinical evidence that chronic heavy alcohol consumption exacerbates skeletal muscle pathology in patients with peripheral artery disease (PAD). PAD patients with alcohol use disorder (AUD) demonstrated significantly greater intramuscular oxidative stress, mitochondrial dysfunction, and myofiber degeneration, which were associated with pronounced impairments in walking capacity and muscle strength.

Importantly, our findings implicate aldehyde dehydrogenase 2 (ALDH2) as a potential mechanistic mediator of alcohol-exacerbated myopathy. ALDH2 downregulation in PAD-AUD muscle coincided with increased acetaldehyde and 4-HNE accumulation, suggesting impaired detoxification of reactive aldehydes. Even in PAD patients without significant alcohol use, elevated aldehyde burden alongside insufficient ALDH2 upregulation may contribute to muscle oxidative damage. These observations point to ALDH2 not only as a biomarker of aldehyde stress but also as a promising therapeutic target for mitigating muscle injury in PAD, irrespective of alcohol use status.

Future studies should aim to clarify the causal role of ALDH2 in mediating ischemic muscle degeneration and evaluate whether pharmacologic activation or gene-based modulation of ALDH2 can restore redox balance and improve functional outcomes in PAD. Screening for and addressing alcohol misuse in this population should also be prioritized as part of comprehensive PAD management.

## Supplementary Information

Below is the link to the electronic supplementary material.

ESM 1 Supplementary Material 1 (PDF 8.28 MB)

## Data Availability

The data that support the findings of this study are available from the corresponding author upon reasonable request.
