# Supplementary material for: Heavy alcohol use exacerbates skeletal myopathy in peripheral artery disease
Source: GeroScience. 2025 Nov 12;48(4):5895–911. doi: 10.1007/s11357-025-01965-3 (PMC13575007; doi:10.1007/s11357-025-01965-3)

4-HNE

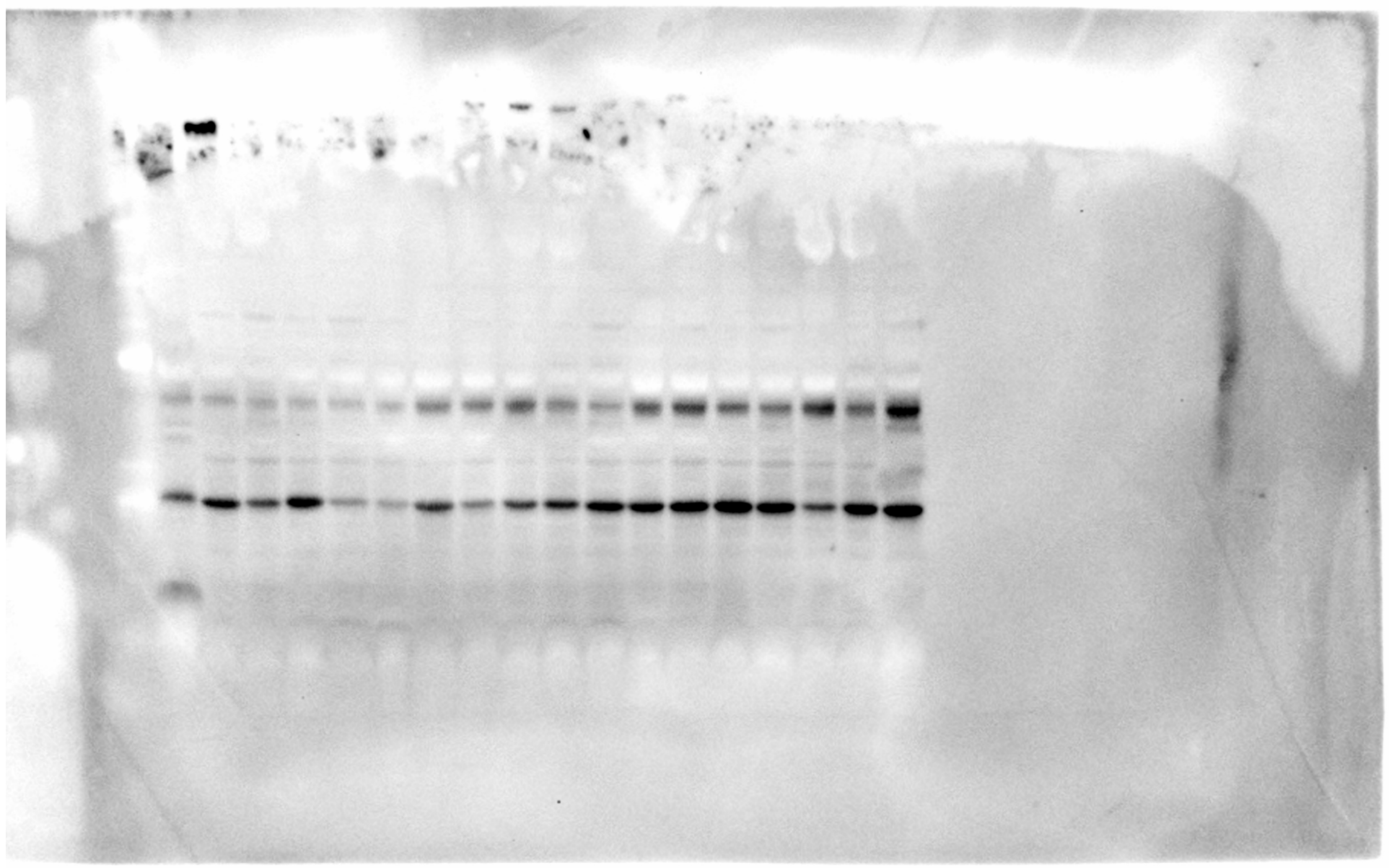

Ponceau

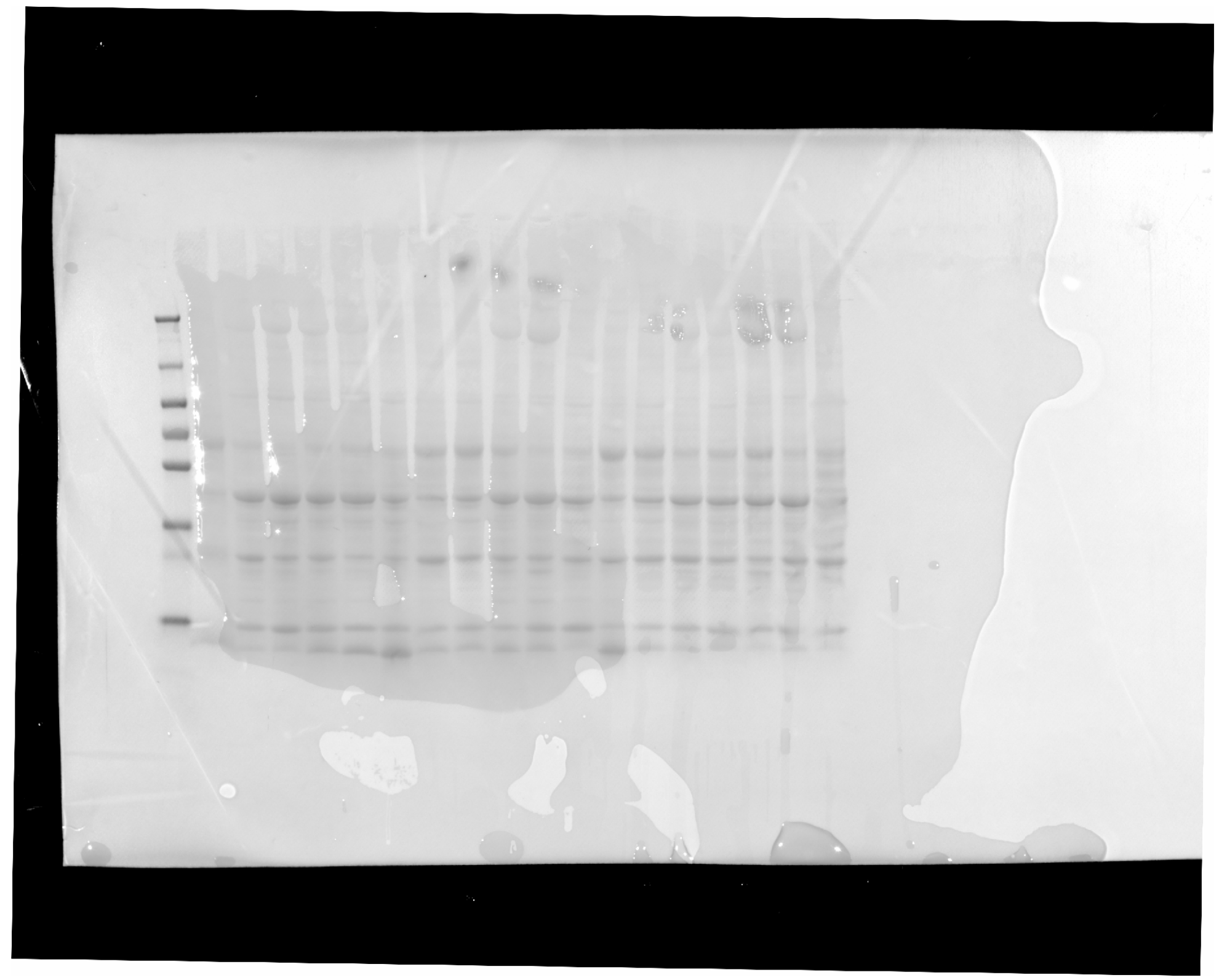

ADH1b

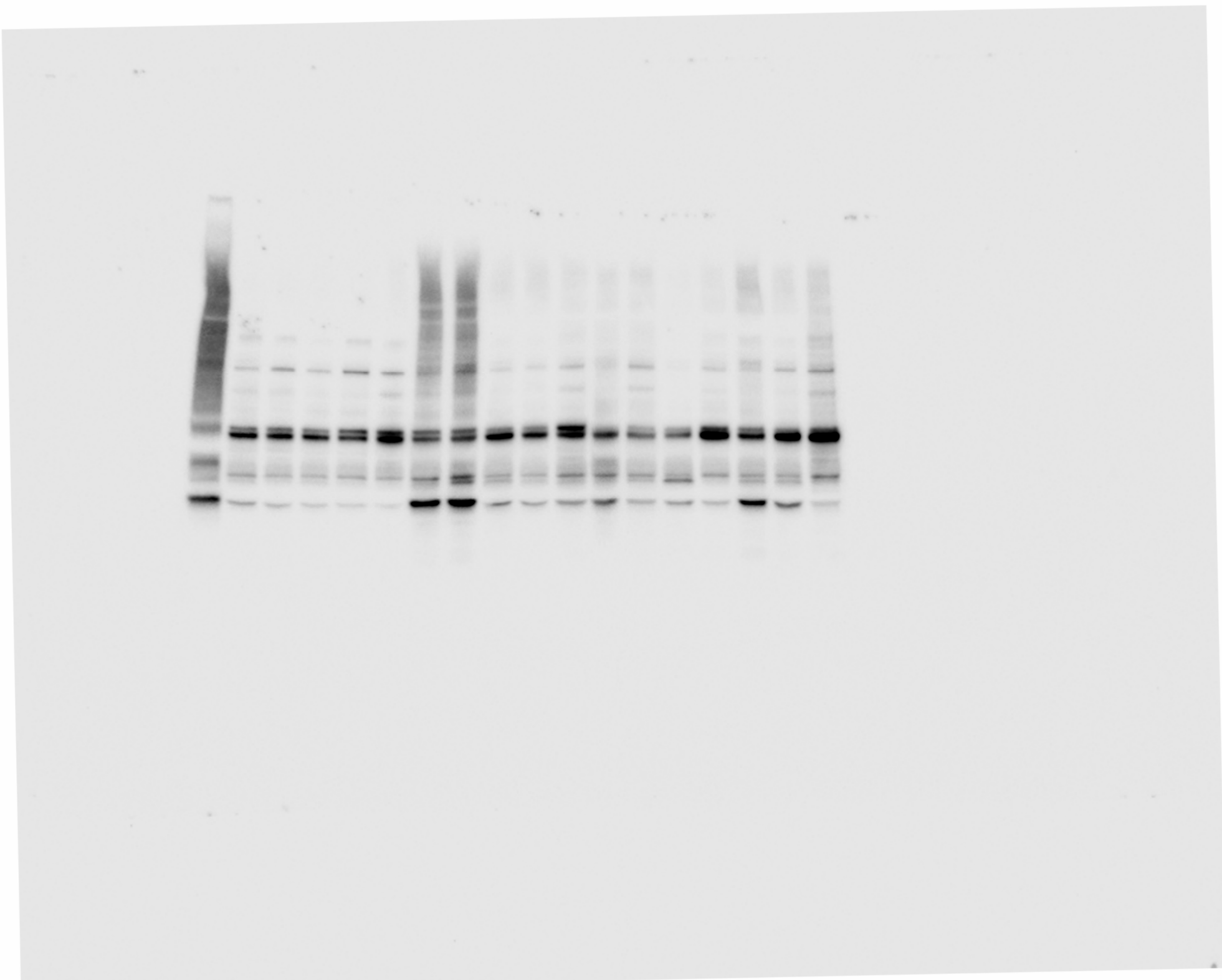

Ponceau

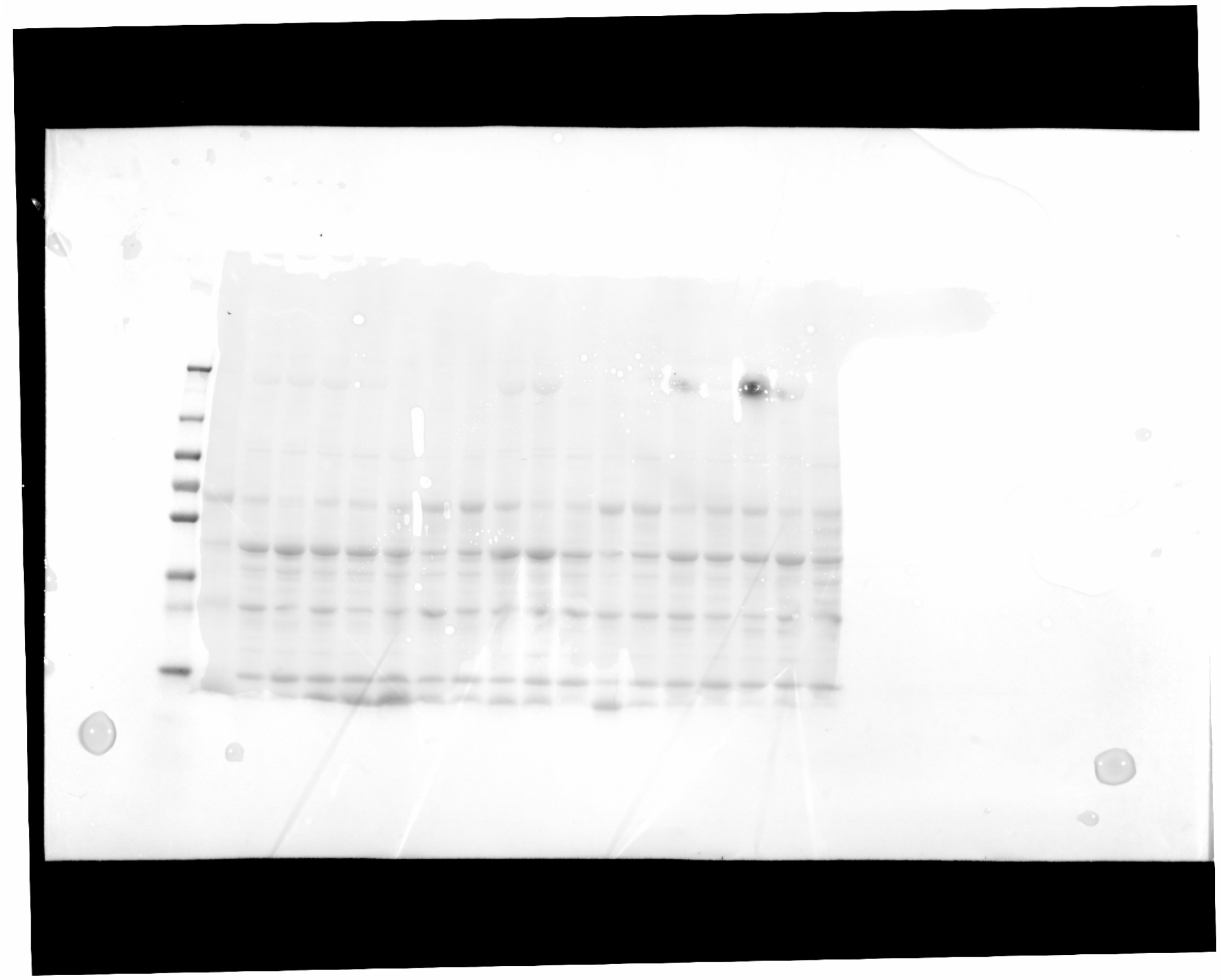

ALDH2

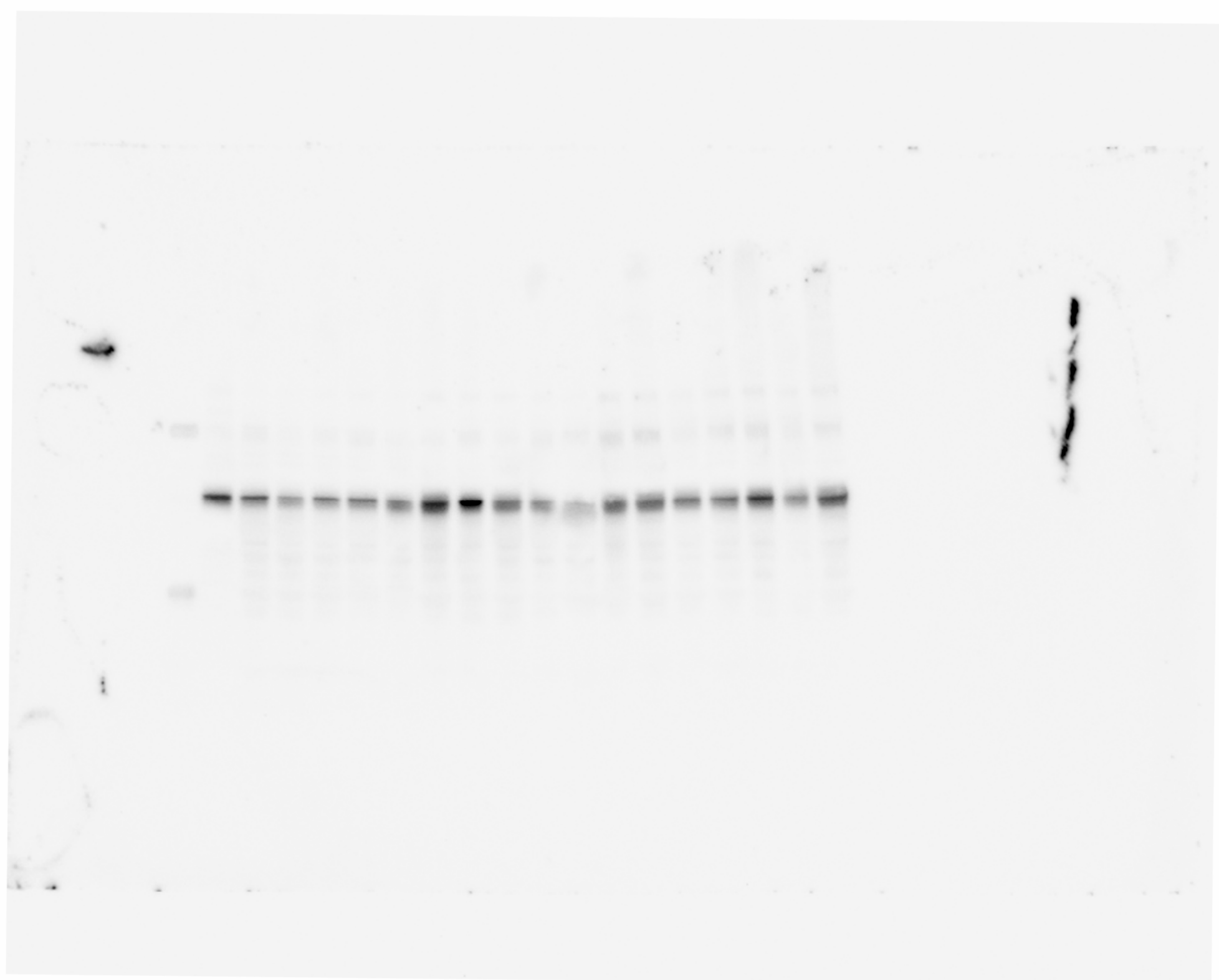

Ponceau

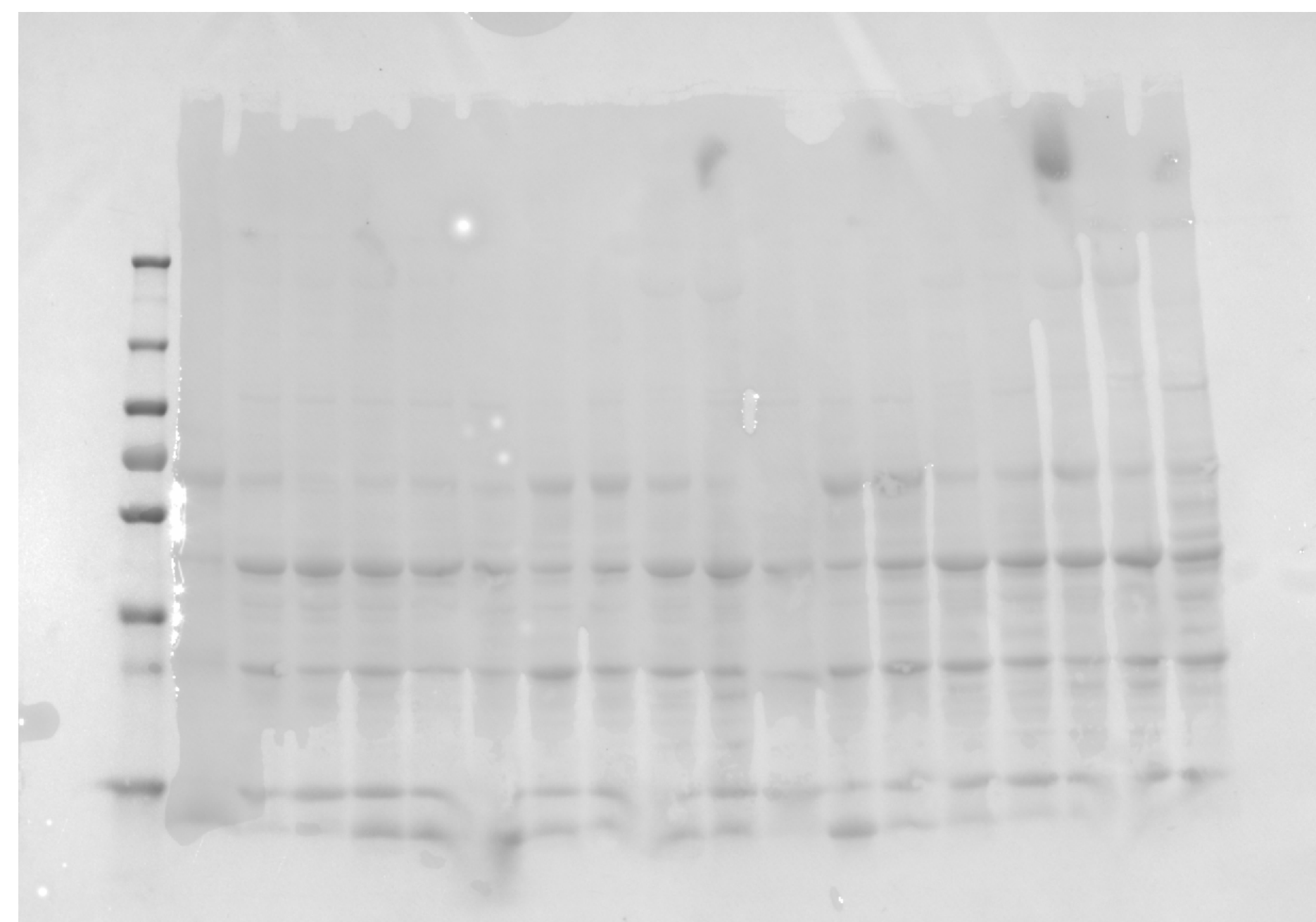

Carbonyl

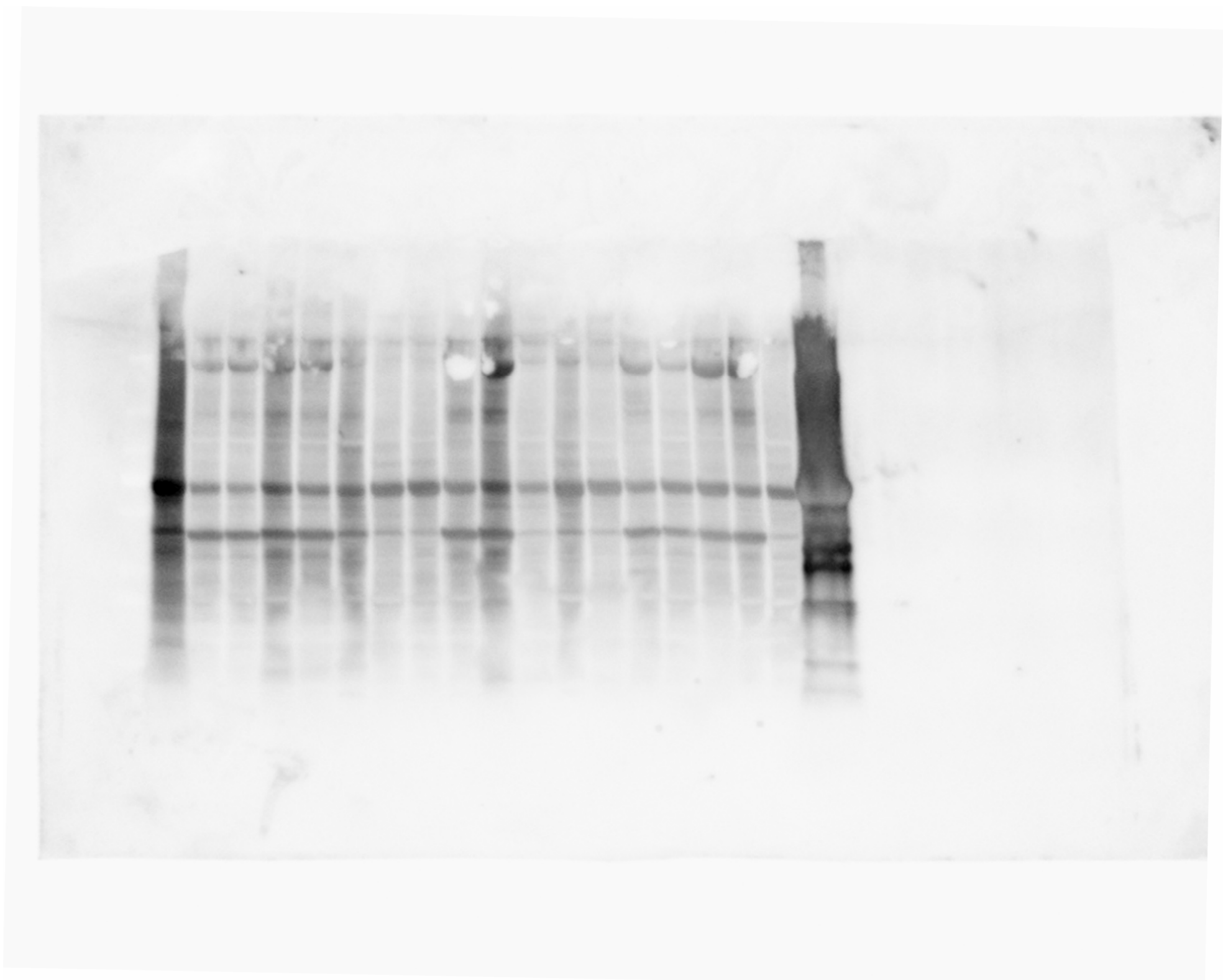

Ponceau

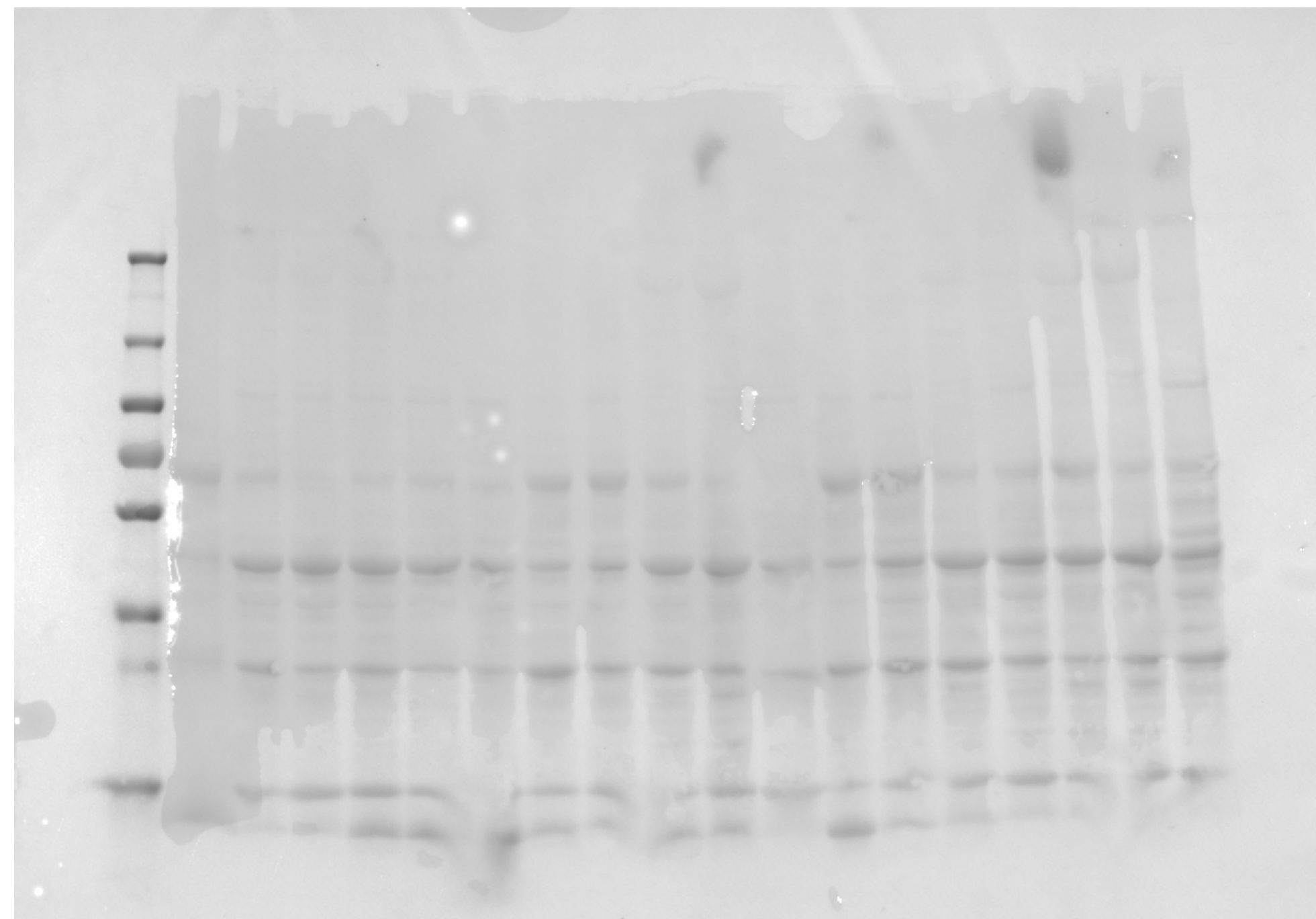

OXPHOS

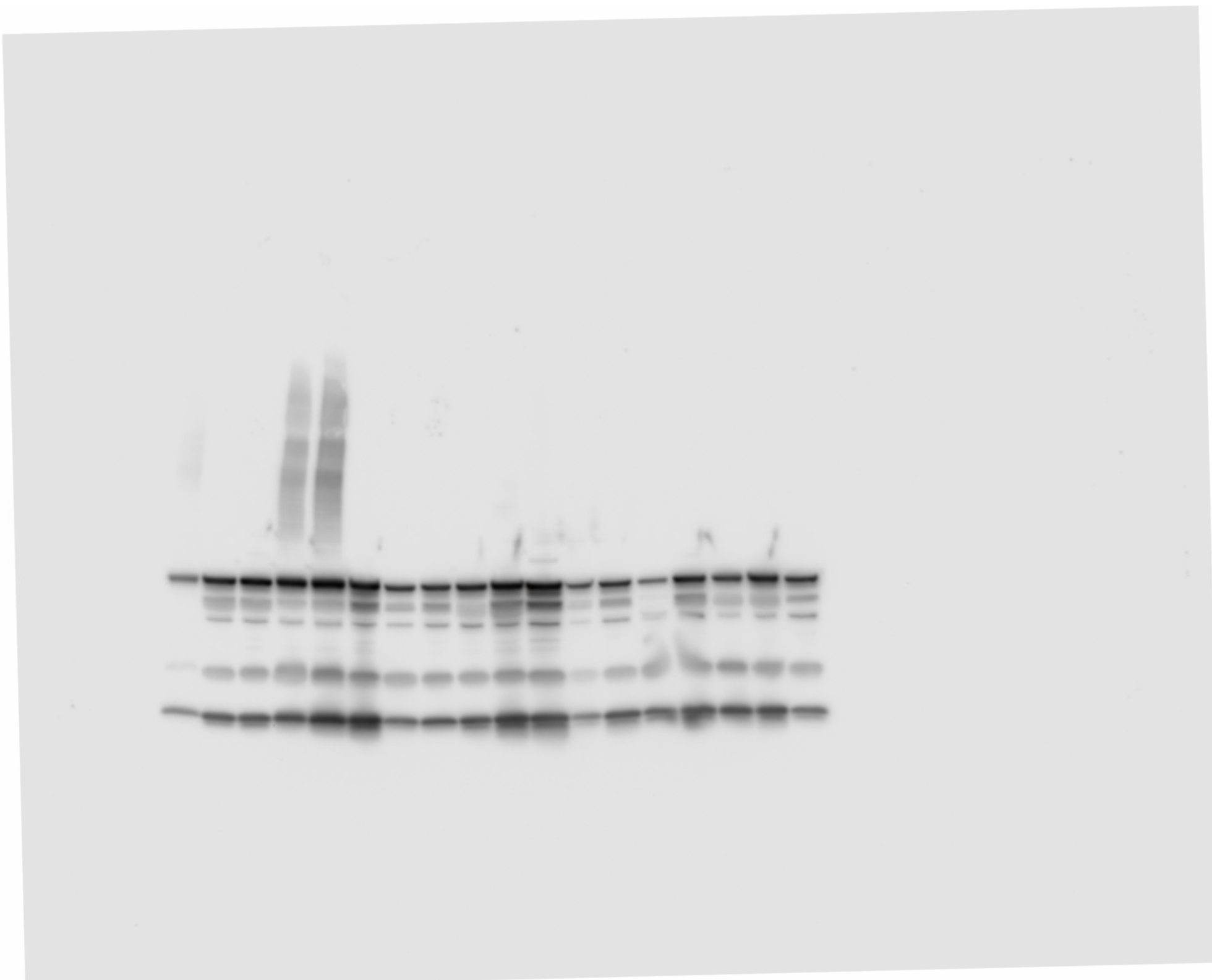

Ponceau

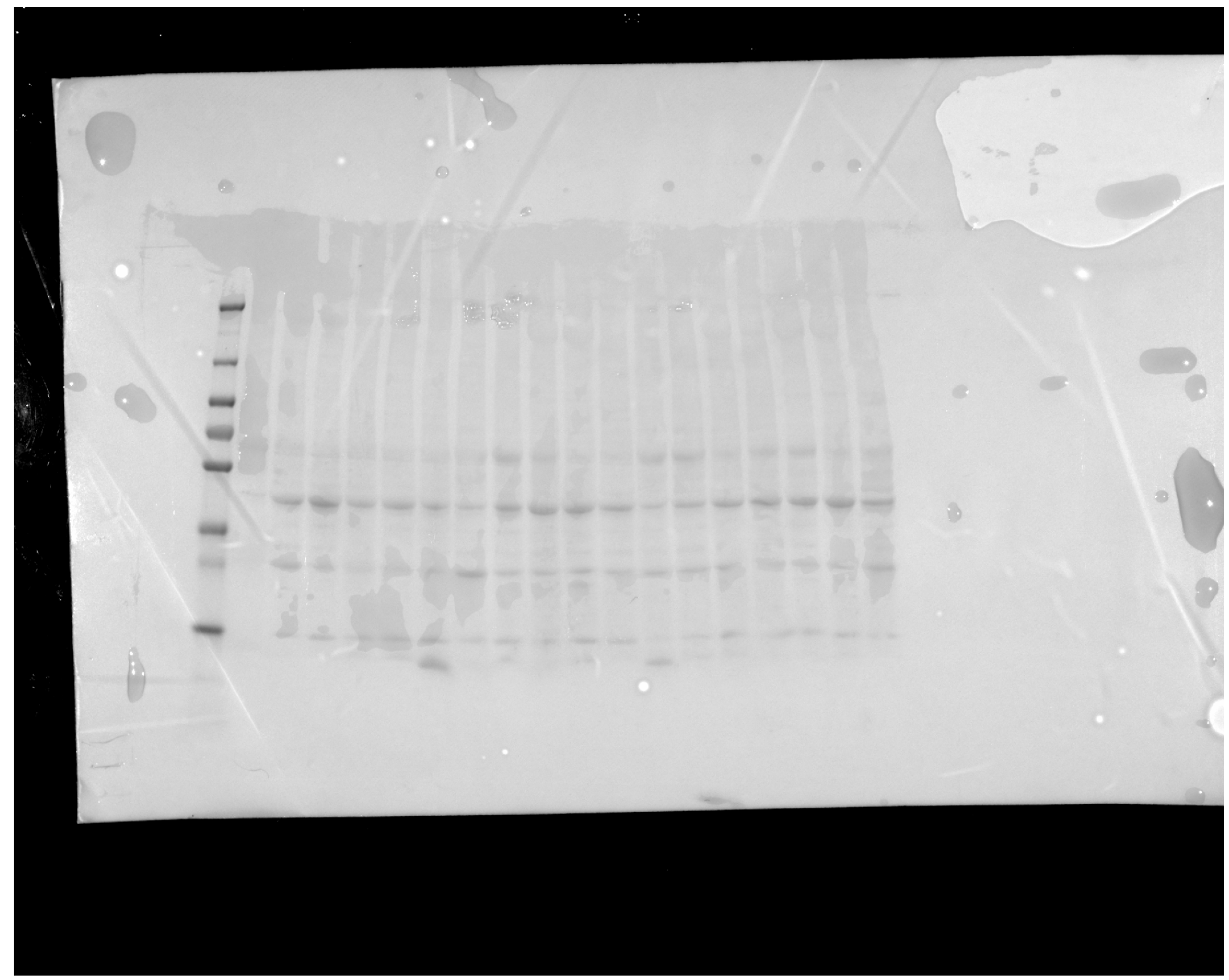

Supplement: Supplementary file 1 [file 11357_2025_1965_MOESM1_ESM.pdf]
